# Formaldehyde exposure induces regulatory T cell-mediated immunosuppression via calcineurin-NFAT signalling pathway

**DOI:** 10.1038/s41598-020-72502-9

**Published:** 2020-10-12

**Authors:** Jeongsik Park, Hyo-Seon Yang, Mi-Kyung Song, Dong Im Kim, Kyuhong Lee

**Affiliations:** 1 Jeonbuk Department of inhalation Research, National Center for Efficacy Evaluation of Respiratory Disease Products, Korea Institute of Toxicology, 30 Baehak 1-gil, Jeonguep, Jeollabuk-do 56212 Republic of Korea; 2 Department of Human and Environmental Toxicology, University of Science and Technology, Daejeon, 34113 Republic of Korea

**Keywords:** Cytokines, Lymphocytes

## Abstract

In this study, we investigated the effects of Formaldehyde (FA) exposure on splenic immune responses wherein helper T cells become activated and differentiate into effector T and regulatory T cells. BALB/c mice were exposed to two FA concentrations (1.38 mg/m^3^ and 5.36 mg/m^3^) for 4 h/day and 5 days/week for 2 weeks. FA-induced immune responses were examined by the production of cytokines, expression of mRNAs, and distributions of helper T cells and regulatory T cells. Moreover, expression of calcineurin and NFATs, regulatory T cell-related signalling proteins, were evaluated. FA exposure suppressed Th2-, Th1-, and Th17-related splenic cytokines in a dose-dependent manner. mRNA expression of splenic cytokines was also decreased by FA exposure, which correlated with decreased cytokine expression. In parallel, FA exposure promoted T cell differentiation into regulatory T cells in a dose-dependent manner supported by the expression of calcineurin and NFAT1. Taken together, our results indicated that FA exposure increases the number of regulatory T cells via calcineurin-NFAT signalling, thereby leading to effector T cell activity suppression with decreased T cell-related cytokine secretion and mRNA expression. These findings provide insight into the mechanisms underlying the adverse effects of FA and accordingly have general implications for human health, particularly in occupational settings.

## Introduction

Formaldehyde, a colourless, highly reactive compound emitted from homes and buildings, is a common indoor air pollutant that induces harmful health effects^1^. The major indoor sources of FA are plywood, particle board, urea-FA foam insulation, floor coverings, and furniture^2^. FA is also found in many products, such as plastics, adhesives, cosmetics, and fungicides^3^. Many individuals are constantly exposed to FA via inhalation and skin absorption at home and/or the workplace.

According to the U.S. Environmental Protection Agency (EPA) toxicological review, FA induces sensory irritation, asthma and atopy, immune system alterations, neurotoxicity, and reproductive and developmental toxicity^4^. Recent human and animal studies have indicated that the immune system may be a target for FA toxicity. FA leads to immune dysregulation or immunosuppression and may consequently cause allergies or cancer progression. FA is a potential allergen for allergic contact dermatitis and asthma^5–8^. The incidence of allergic asthma increases as the indoor FA concentration increases^9,10^. Garrett et al.^11^ found that low-level exposure to indoor FA increases the risk of developing asthma and aggravates atopy symptoms in children. In rodent models, FA exposure induces T lymphocytes and related cytokines as well as allergic airway inflammation^12–14^.

FA could affect the populations of different types of immune cells^15^. For example, it increased the percentage of B cells but decreased the percentages of total T cells (CD3^+^) and cytotoxic T cells (CD8^+^) in the blood of FA-exposed workers, while helper T cells (CD4^+^) remain unchanged^16^. Hosgood et al. ^15^ recently reported that natural killer, CD4^+^, and CD8^+^ cell counts were decreased in FA-exposed workers. There were no differences in lymphocytes or T cells (CD4^+^ and CD8^+^) in individuals exposed to FA during an accidental spill^17^. The inconsistencies among the results of previous studies suggest that further analyses of the effects of FA on the immune system are needed to understand its adverse health effects.

Helper T cells play a central role in modulating immune responses and can be subdivided into two main subsets: effector T cells (Th1, Th2, and Th17) and regulatory T (Treg) cells^18^. These subsets exhibit distinct patterns of cytokine secretion and specific expression of master transcription factors in response to exogenous pathogens.

Th1 cells are characterised by the production of IFN-γ and induce phagocyte activation and the production of opsonising antibodies, thus playing an important role in host defence against intracellular pathogens^19^. Th1 cells also contribute to the development of organ-specific autoimmune diseases and chronic inflammatory disorders^20^. Th2 cells produce IL-4, IL-5, and IL-13 and have a critical role in the differentiation, maintenance, and amplification of Th2-type immune disorders, such as asthma and atopic dermatitis^21,22^. Th17 cells play important roles in the clearance of yeast, fungi, and extracellular bacteria and produce IL-17A and IL-22, which recruit neutrophil granulocytes^23–25^. Th17 cells are also involved in the pathogenesis of autoimmune and inflammatory diseases^26^.

Treg cells constitute a unique helper T cell lineage characterised by the expression of the master transcription factor forkhead box P3 (Foxp3)^27–29^. Foxp3 is essential for their development, maintenance, and suppressive function^27,28^. Treg cells also play a crucial role in immunological tolerance to maintain immune system homeostasis, regulate effector T cell responses, and prevent chronic inflammation and autoimmune diseases^21,24,29^. However, in addition to their beneficial effects, excessive populations of Treg cells have deleterious effects on hosts by suppressing sterilising immunity and limiting antitumor immunity^30^. Cavassni et al.^31^ found significantly increased numbers of Treg cells after recovery from severe sepsis and this was related to increased tumour growth and the inhibition of the antitumor effects of CD8^+^ T cells. Exposure to environmental toxicants, such as arsenic, promotes the differentiation of Treg cells and thus prevents Th1- and Th2-related cytokine production and increases susceptibility to opportunistic infections, such as tuberculosis^32,33^. These findings suggest that increases in Treg cells inhibit effector T cell activity and contribute to the development of immunosuppressive microenvironments, thus resulting in adverse health effects, such as immune evasion and cancer progression^34–36^.

Many studies have reported a relationship between FA exposure and helper T cells^12,16,17,37,38^. However, the role of regulatory T cells in immune modulation induced by FA remains poorly understood.

In the present study, we examined the effects of FA exposure on the immune system via the regulation of the T cell population and distinct patterns of cytokine expression by evaluating the distributions of helper T cells and regulatory T cells in the spleen of an FA-exposed mouse model. In addition, the expression levels of Th1 (IFN-γ), Th2 (IL-4, IL-5, and IL-13), and Th17 (IL-17A, and IL-22)-related cytokines in FA-exposed mice were evaluated.

## Results

### Changes in body weight gains and organ weights of FA-exposed mice

**Figure 1:**
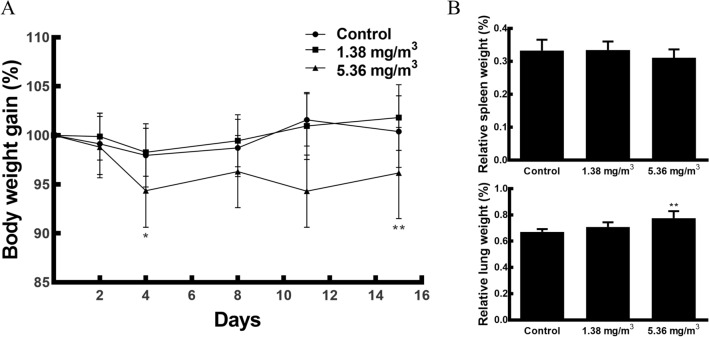
Changes in body weight gains and organ weights of FA-exposed mice. (**A**) Changes in the body weight gains in FA-induced mice during the experimental period. (**B**) Relative spleen and left lung weights were calculated as the ratio of organ weight to body weight. Data are presented as means ± SD (*n* = 10 mice/group). *p < 0.05 versus control group, **p < 0.01 versus control group.

Figure 1A shows the effect of FA exposure on the body weight gains of mice. There was no significant difference in body weight gain between the 1.38 mg/m^3^ FA exposure group and the control group. However, exposure to 5.36 mg/m^3^ FA resulted in a marked decrease in body weight gain on day 4 and day 15. Figure 1B summarises the effect of FA exposure on relative organ weights (organ weight/body weight × 100%) in mice. There were no significant differences in relative spleen weight between FA exposure groups and the control group. However, the relative lung weight was significantly higher in the 5.36 mg/m^3^ FA exposure group than in the control group.

### Effects of FA exposure on Con A-induced splenic cytokine production and mRNA expression

**Figure 2:**
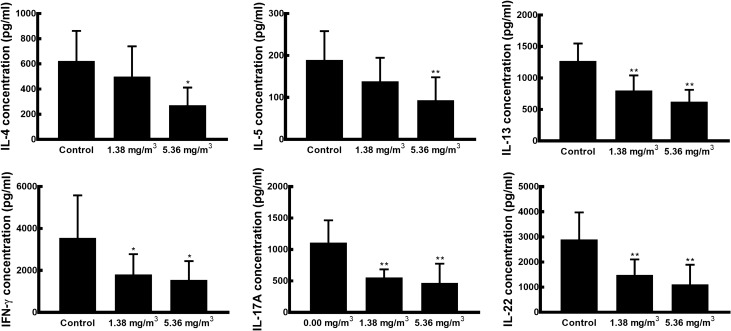
Effects of FA exposure on Con A-induced splenic cytokine production. Spleen cells isolated from mice were incubated in a 5% CO_2_ incubator at 37 °C for 48 h in the presence of Con A. Production of Th2 (IL-4, IL-5, and IL-13)-, Th1 (IFN-γ)-, and Th17 (IL-17A and IL-22)-related cytokines in the spleen cell culture supernatant was measured using a multiplex Luminex system. Data are presented as means ± SD (*n* = 10 mice/group). *p < 0.05 versus control group, **p < 0.01 versus control group.

**Figure 3:**
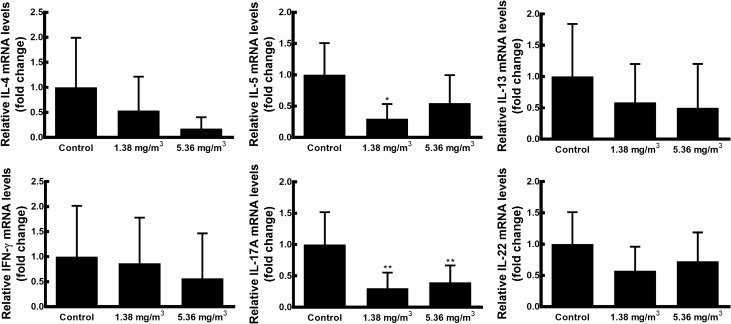
Effects of FA exposure on Con A-induced splenic cytokine mRNA expression levels. Spleen cells isolated from mice were incubated in a 5% CO_2_ incubator at 37 °C for 48 h in the presence of Con A. Levels of Th2 (*IL-4*, *IL-5*, and *IL-13*)-, Th1 (*IFN-γ*)-, and Th17 (*IL-17A* and *IL-22*)-related mRNAs in the spleen cell were measured by qRT-PCR. Gene expression was normalised using *GAPDH* expression and results are presented as fold changes relative to the control group. Data are presented as means ± SD (*n* = 10 mice/group). *p < 0.05 versus control group, **p < 0.01 versus control group.

To investigate the effect of FA exposure on the immune response, Th2 (IL-4, IL-5, IL-13)-, Th1 (IFN-γ)-, and Th17 (IL-17, IL-22)-related cytokines and mRNAs were measured in the Con A-stimulated spleen cell culture supernatant (Fig. 2). All splenic cytokines were produced in a dose-dependent manner, and cytokine production in the 5.36 mg/m^3^ FA exposure group was significantly lower than that in the control group. Decreased mRNA expression levels were correlated with decreased cytokine production, and the mRNA expression levels of *IL-5* and *IL-17A* in the FA exposure groups were significantly lower than those in the control group (Fig. 3).

### Effects of FA exposure on splenic helper T cells and Treg cells

**Figure 4:**
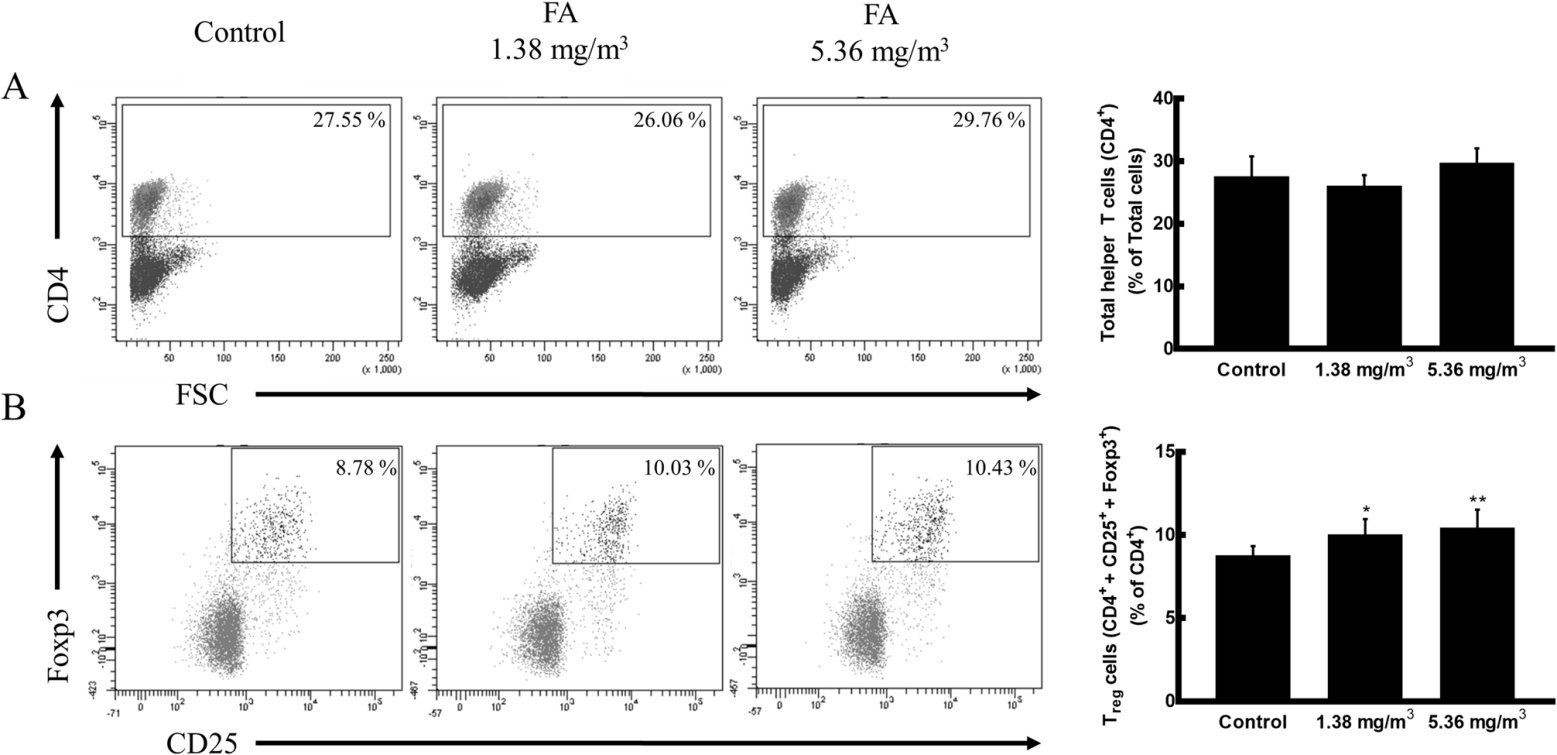
Effects of FA on splenic helper T cells and Treg cells. Spleen cells isolated from mice were stained with an FITC-conjugated anti-CD4 monoclonal antibody, APC-conjugated anti-CD25 monoclonal antibody, and PE-conjugated anti-Foxp3 monoclonal antibody. FITC, APC, and PE fluorescence levels were measured by flow cytometry. (**A**) Populations of CD4^+^ T cells in spleen cells (expressed as representative dot plots and percentages). (**B**) Populations of CD4^+^ CD25^+^ Foxp3^+^ T cells in CD4^+^ T cells (expressed as representative dot plots and percentages). Data are presented as means ± SD (*n* = 10 mice/group). *p < 0.05 versus control group, **p < 0.01 versus control group.

To confirm whether the decrease in T cell-related cytokines is related to a change in the T cell population in the spleen, flow cytometry was performed after gating the helper T cell population. There was no difference in the percentage of CD4^+^ helper T cells among groups (Fig. 4A). However, the Treg cell population (CD4^+^ CD25^+^ Foxp3^+^) increased significantly from 8.78 to 10.03% and 10.43% following exposure to 1.38 mg/m^3^ and 5.36 mg/m^3^ FA, respectively (Fig. 4B).

### Effect of FA exposure on the activation of calcineurin-NFAT signalling in the spleen

**Figure 5:**
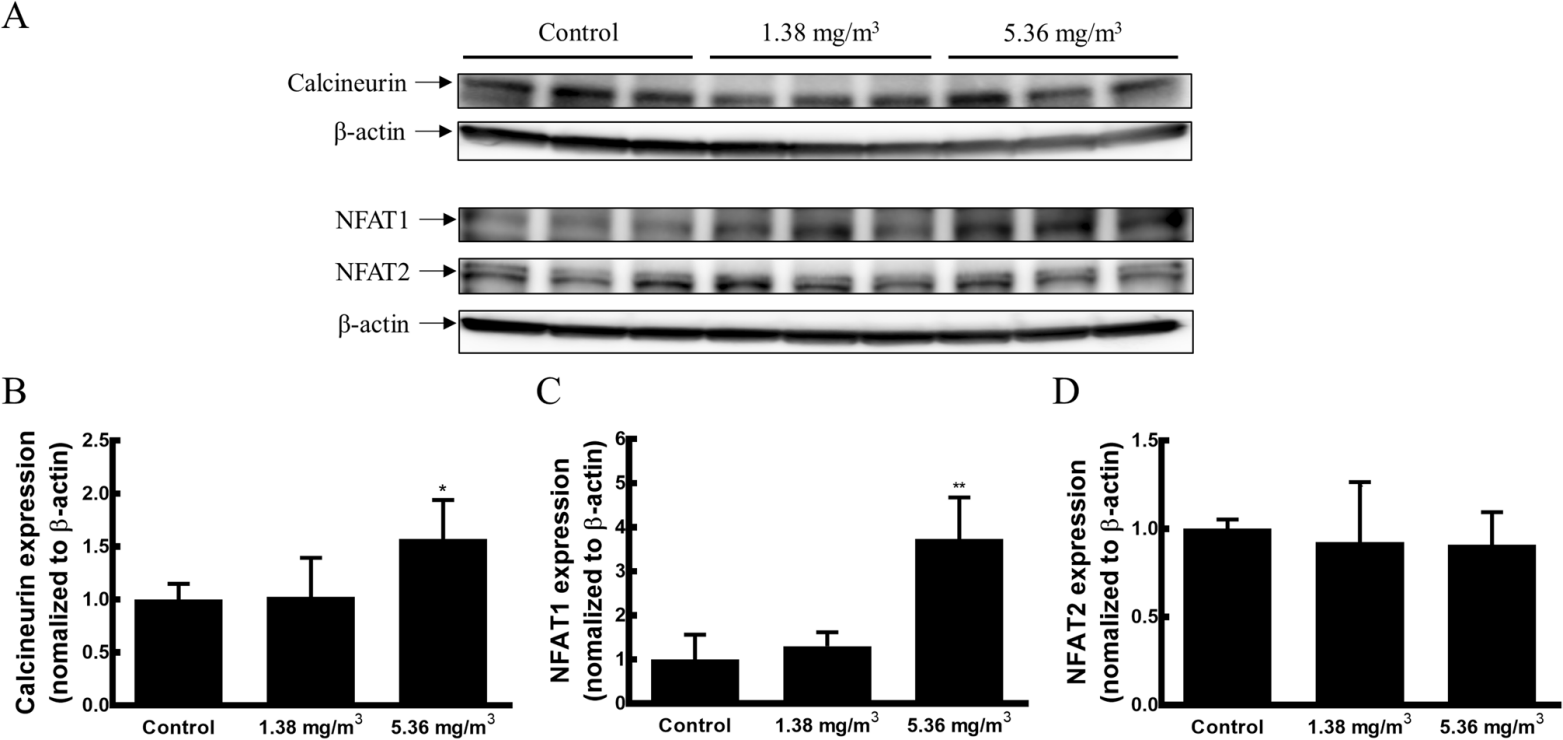
Effect of FA exposure on the activation of calcineurin-NFAT signalling in the spleen. (**A**) Calcineurin, NFAT1, and NFAT2 levels in the spleen were detected by western blotting. (**B**) The blots were analysed by densitometry and levels were normalised against β-actin. Values are presented as means ± SD (*n* = 6 mice/group). *p < 0.05 versus control group, **p < 0.01 versus control group.

To elucidate the molecular mechanism by which the Treg cell population changes in response to FA exposure, we evaluated the protein expression of calcineurin, NFAT1 and NFAT2, which are well-known T cell receptor (TCR) signalling proteins related to the regulation of Treg cell development. The protein expression of NFAT1 in the 5.36 mg/m^3^ FA exposure group was approximately 3.73-fold higher than that in the control group (Fig. 5C). These elevated NFAT1 expression levels were correlated with increased calcineurin expression levels (approximately 1.57-fold higher than those in the control group) (Fig. 5B). NFAT2 expression in the FA exposure group was decreased by approximately 0.91-fold compared with the level in the control group (Fig. 5D). These data suggested that FA exposure induced the differentiation of Treg cells via a calcineurin-NFAT- mediated TCR signalling pathway.

## Discussion

In this study, we found that FA exposure suppresses the splenic immune response, as evidenced by changes in the distribution of helper T cells and expression levels of immune-related factors, including cytokines, mRNAs, and proteins, in BALB/c mice.

FA is a common indoor air pollutant absorbed by inhalation and the skin. In many etiological and toxicological studies, it has been demonstrated about the adverse effects of FA exposure via these routes^2,5–14,39–41^. A concentration of 1.38 mg/m^3^ FA was used in the present study based on the no-observed-adverse-effect concentration (NOAEC, 2.46 mg/m^3^) for mice according to the Organisation for Economic Co-operation and Development Screening Information Dataset (OECD SIDS)^42^. A similar concentration of indoor FA is often found in occupational settings. Tang et al.^43^ reported that the indoor FA concentration is 1.37 mg/m^3^ in factories and 1.46 mg/m^3^ in anatomy and pathology laboratories in medical colleges and hospitals. A concentration of 5.36 mg/m^3^ FA was also evaluated; this is the lowest observed adverse effect concentration (LOAEC, 5.04 mg/m^3^) for mice according to OECD SIDS^42^. Previous animal studies have shown that treatment with FA at the LOAEC induces immunological changes at the proteomic and genomic levels^44,45^. Based on these reports, we used FA concentrations between 1 and 5 mg/m^3^ to evaluate the effects of FA exposure on immune systems in mice.

Our results showed that 5.36 mg/m^3^ FA induces a significant decrease in body weight gain; however, there were no significant differences between the 1.38 mg/m^3^ FA exposure group and the control group. These findings are consistent with those of previous studies^12,39^ and indicate that FA could act as a major indoor air toxicant. We checked lung weight and infiltration of inflammatory cells to evaluate toxicological responses in lung by inhalation route of FA. Our results showed that the relative lung weight was significantly higher in the 5.36 mg/m^3^ FA exposure group than in the control group. In fact, Lino-dos-Santos-Franco et al.^46^ reported that FA exposure cause a significant increase of pulmonary vascular permeability. These results may be related to increase relative lung weight of 5.32 mg/m^3^ FA-exposed mice. However, there were no significant increases in various types of inflammatory cells, including macrophages, eosinophils, neutrophils, and lymphocytes, in BAL fluid (data not shown). These findings are consistent with a previous study showing that FA induces no difference in cell recruitment into the lung^39^. Additionally, in a histopathological analysis, inflammatory cell infiltration was slightly increased in the 5.36 mg/m^3^ FA exposure group, with a positive rate of 20% (2/10, data not shown). The FA concentrations used in this study only induce a minor inflammatory response in the lung tissues of some individuals but do not induce direct lung injury. In the present study, we examined immunological changes in the spleen at FA concentrations that have no direct impact on the lungs.

The spleen is a highly systematic lymphoid organ in which adaptive immune responses can be initiated^47^. In the spleen, T lymphocytes become activated and differentiate into Th1, Th2, Th17, and Treg cells by the production of corresponding effector cytokines^21^. In humans and various animal models, FA exposure has been found to alter immune responses either by activation or suppression, which may lead to changes in the number of helper T cells and cytokine production^14,37,38^. Thus, we evaluated the effect of FA exposure on helper T cell-related cytokines and mRNAs using Con A-, inducing mitogenic activity of T lymphocytes and increasing synthesis of cellular products, activated spleen cells. Our results show that FA exposure suppresses the production of all splenic cytokines in a dose-dependent manner and cytokine production in the 5.36 mg/m^3^ FA exposure group was significantly lower than that in the control group. This result corresponded to the observed decreases in mRNA expression levels in FA-exposed mice. These findings are consistent with the results of previous studies reporting suppressed production of Th1- and Th2-related cytokines in FA-exposed rodent models with ovalbumin sensitisation, thus leading to a decrease in the development of allergic lung inflammation^13,39^. Furthermore, recent studies have revealed that FA exposure suppressed the cytokine production and mRNA expression of IFN-γ in C57BL/6 mice^48^ and BN rats^49^. And Wei et al.^14^ found that Th2-, Th1-, Th17-related cytokine showed the tendency to be depressed in FA-exposed C57BL/6 mice. These observations highlighted that FA exposure inhibits effector T cell activity, resulting in decreases in T cell cytokine secretion and mRNA expression.

Treg cells are a distinct T lymphocyte lineage endowed with regulatory functions that affect a variety of immune cells in innate and adaptive immunity as well as the priming and effector phases of immune responses^50–52^. The various potential suppressive mechanisms can be divided into four general categories as follows: suppression by inhibitory cytokines, suppression by cytolysis, suppression by metabolic disruption, and suppression by the modulation of dendritic cell maturation or function^30^. Therefore, to investigate whether FA exposure can induce immunosuppression, we examined the distribution of Treg cells and their signalling pathways. To our knowledge, this is the first report demonstrating the effect of FA exposure on the differentiation of Treg cells in a mouse model. In the present study, we found that FA exposure increases the number of splenic CD4^+^ CD25^+^ Foxp3^+^ Treg cells in a dose-dependent manner. The Treg cell population in the 5.36 mg/m^3^ FA exposure group was significantly higher (by approximately 20%) than that in the control group. In contrast, the percentage of CD4^+^ helper T cells was not affected by FA exposure, consistent with previous studies of FA-exposed humans as well as mouse models^12,16,17^.

Recent studies have reported that the calcineurin-NFAT-mediated TCR signalling pathway has crucial roles in the differentiation, maintenance, and suppressive function of Treg cells by inducing Foxp3 expression and interacting with NFAT and Foxp3^29,53–55^. The NFAT family includes 5 members, NFAT1 to NFAT5, of which NFAT1 and NFAT2 are preferentially expressed in peripheral T cells^56,57^. NFATs are dephosphorylated by activated calcineurin, which leads to their nuclear translocation and the induction of NFAT-mediated gene transcription^58,59^. Interestingly, individual NFAT gene-deficient mice develop quite disparate phenotypes^60^. NFAT1-deficient mice have lymphoid hyperplasia with hyperproliferation and elevated levels of IL-4, IL-5, and IgE production^61–64^. In contrast, NFAT2-deficient mice show reduced numbers of thymocytes and impaired proliferation of effector T cells, thus leading to decreases in IL-4 and IL-17A production^65–67^. These findings indicated that NFAT1 has regulatory or inhibitory functions, while NFAT2 promotes the proliferation of effector T cells and cytokine production in the immune system. It has been reported that NFAT1 plays an important role in enhancing Foxp3 expression^68,69^, maintaining stable Foxp3 expression^70^, and the suppressive function^54,55,71^ of Treg cells. Furthermore, Foxp3 inhibits activation-induced NFAT2 expression in T cells, thereby limiting effector cytokine expression^72,73^. Our results show that FA exposure increases NFAT1 expression in a dose-dependent manner. NFAT1 expression was significantly higher in the 5.36 mg/m^3^ FA exposure group than in the control group. Increased NFAT1 expression was correlated with increased calcineurin expression in FA-exposed mice. FA exposures resulted in slightly lower NFAT2 expression than that in the control group. These results suggest that FA exposure induces calcineurin-NFAT signalling activation with divergent expression of NFAT1 and NFAT2, thus increasing regulatory T cells and the subsequent development of the immunosuppressive environment.

In the present study, we evaluated the effects of FA on helper T cell-mediated immune responses in mice. Our results indicated that FA exposure suppresses effector T cell activity, with decreased T cell-related cytokine secretion and mRNA expression. FA exposure also induced the differentiation of Treg cells via calcineurin-NFAT signalling activation, which may play crucial roles in the progression of Foxp3-induced immunosuppression. These findings suggest that FA results in the development of an immunosuppressive environment by upregulating the Treg population via calcineurin-NFAT signalling, thereby suppressing effector T cell activities. Furthermore, the differentiation of Treg cells via calcineurin-NFAT signalling activation with divergent expression of NFAT1 and NFAT2 provides insight into the molecular mechanism underlying the immune responses of helper T cells. Although we were unable to confirm the disease-related effects, our results suggest that FA-induced immunosuppression has a pivotal role in the sensitivity to multiple chemicals, opportunistic infections, or cancer progression.

## Methods

### Animals

Five-week-old female BALB/c mice were purchased from Orient Bio Inc. (Seongnam, Korea). All mice were housed in ventilated polypropylene cages in an animal room with controlled temperature (22 ± 3 °C), humidity (50 ± 20%), air ventilation (10–20 times/h), and a 12-h light/dark cycle. All animals were given a sterilized pellet food (PMI Nutrition International, Richmond, IN, USA) and sterilized tap water ad libitum and acclimatized for one week before FA exposure started. All experimental protocols for the study were approved by the Institutional Animal Care and Use Committee of Korea Institute of Toxicology (IACUC #1512-0397). In addition, all methods were performed in accordance with the relevant guidelines and regulations.

### Experimental groups

Mice were randomly divided into three groups (n = 10): control group, 1.38 mg/m^3^ FA exposure group, and 5.36 mg/m^3^ FA exposure group. Control mice were treated in the same chamber but without FA exposure. Mice in treatment groups were exposed to FA for 2 weeks at 4 h/day and 5 days/week in a whole-body exposure chamber. Body weights were measured on days 2, 4, 8, and 11 prior to FA exposure. At 24 h after the last FA exposure, the terminal body weight was measured and mice were sacrificed under isoflurane anaesthesia. Tissue samples were collected for subsequent analyses.

### FA exposure

**Figure 6:**
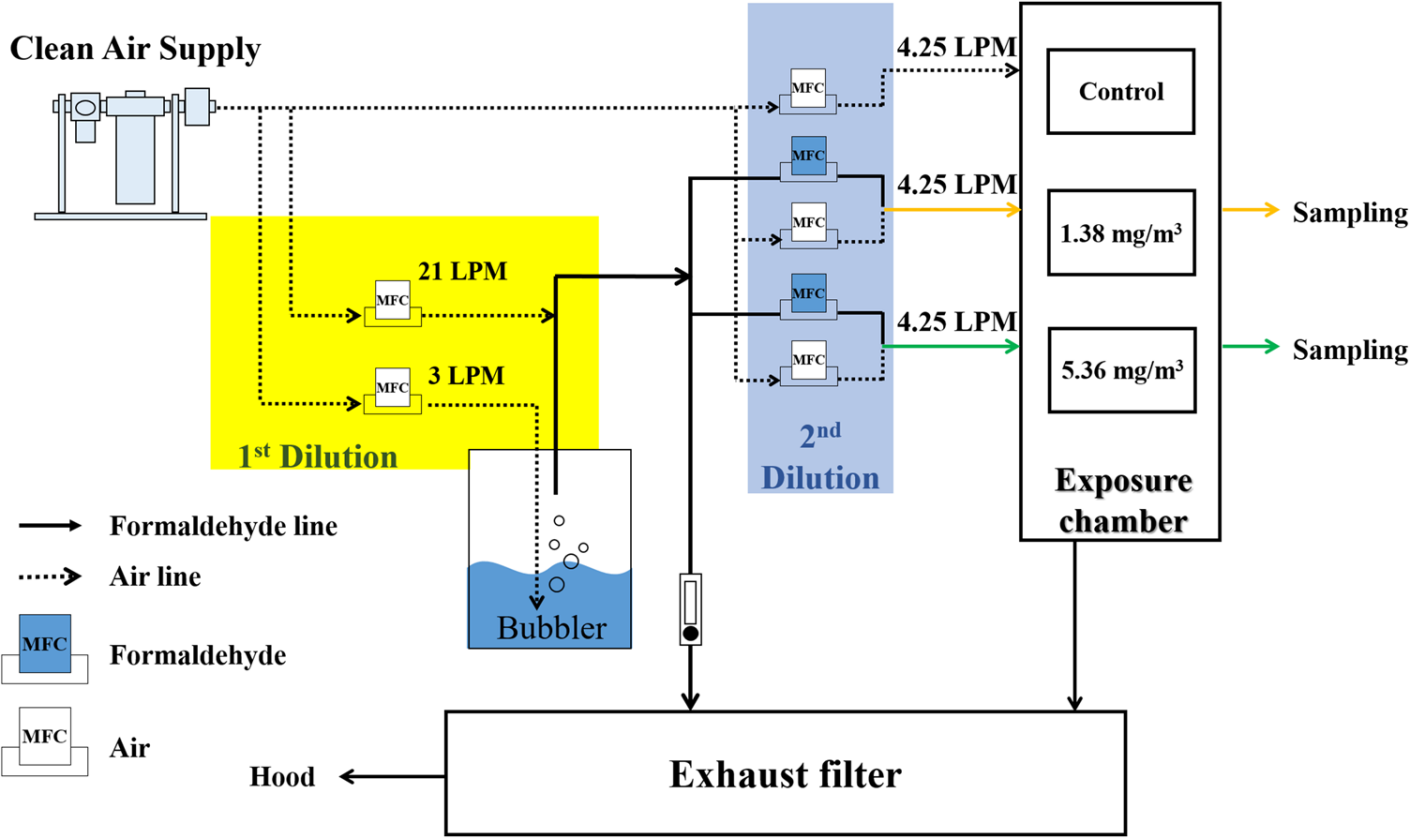
Schematic diagram of generator system for FA exposure. FA was generated using a gas bubbler and mass flow controller. FA concentration, temperature, and humidity in the exposure chamber were monitored by HPLC, and by using thermometer and hygrometer.

FA was generated from a methanol-free 10% FA solution (Polysciences Inc., Warrington, PA, USA) using a gas bubbler and mass flow controller (Fig. 6). It was diluted with clean, filtered air to achieve the desired FA concentrations and delivered to 17-L polycarbonate exposure chambers. FA in the chambers was sampled in a Top Solid DNPH Cartridge (Top-Trading Co., Seoul, Korea) and was monitored hourly by high-performance liquid chromatography.

### HPLC-UV analysis

**Figure 7:**
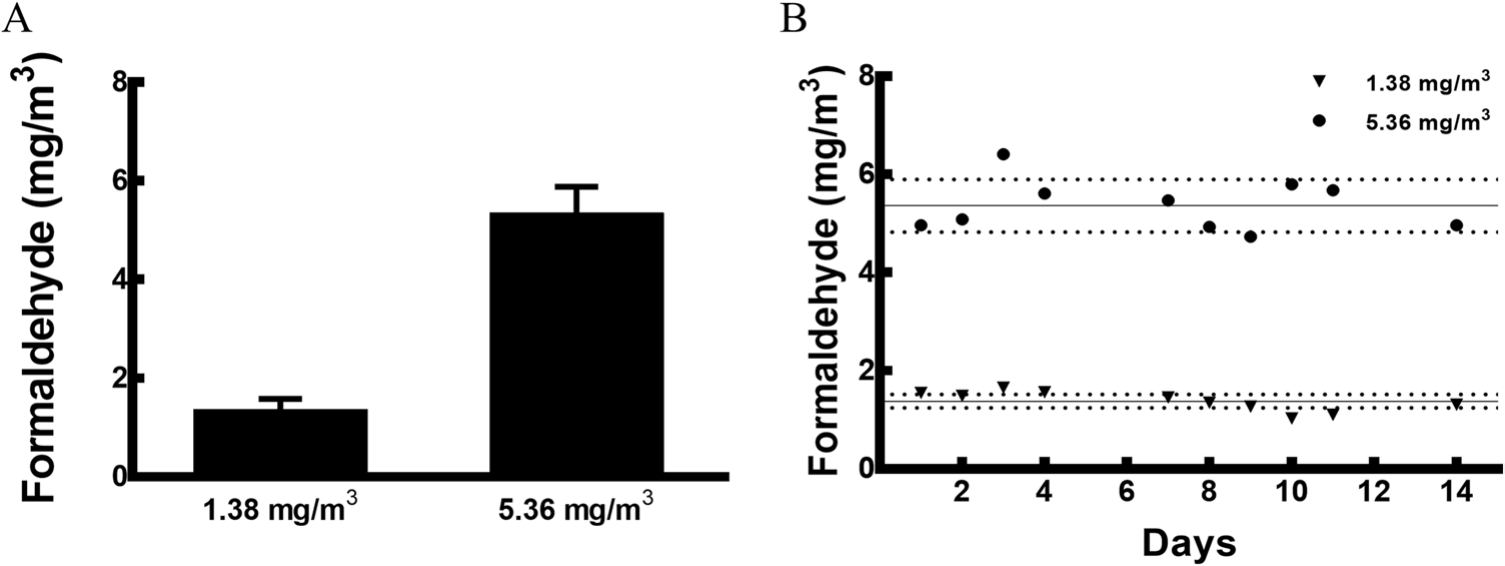
Concentrations of FA. The (**A**) mean and (**B**) daily FA concentrations were monitored using a 2, 4-DNPH cartridge and HPLC–UV. Female BALB/c mice were exposed to two different concentrations (1.38 mg/m^3^ and 5.36 mg/m^3^) of FA for 4 h/day and 5 days/week, for 2 weeks. Data are presented as means ± SD.

The separation and determination of FA-2, a 4-DNPH derivative were performed on an LC-20A HPLC system (Shimadzu, Kyoto, Japan) equipped with binary LC-20AD pumps, a DGU-20A_3_ degasser, a SPD-20A ultraviolet detector, a SIL-20A autosampler, and a CTO-20AC column oven. A Gemini 5u C18 110A column (150 mm 4.6 mm I.D., 5 μm) (Phenomenex, Torrance, CA, USA) was used as an analytical column. The mobile phase of acetonitrile-distilled water (60:40, v/v) was used. The flow rate was 1.0 mL/min. The column temperature was 40 °C and the injection volume was 10 μL. The analyte was monitored at the wavelength of 360 nm. Quantitation was performed using synthesised FA-2, 4-DNPH solution (Sigma-Aldrich Co., St. Louis, MO, USA) as standard. Each group of ten mice was exposed to 0 (control), 1.38 ± 0.20 mg/m^3^ (mean ± SD), or 5.36 ± 0.52 mg/m^3^ FA for 4 h a day and 5 days a week over a 2-week period (Fig. 7).

### Spleen cell preparation and culture

The spleen was removed from each FA-exposed mouse. Single cell suspensions were obtained by mincing the spleen and gently pressing the fragments through a 45-μm nylon cell strainer (BD Falcon, Bedford, MA, USA). The suspension was mixed with 1 × RBC Lysis buffer (eBioscience Inc., San Diego, CA, USA) for 5 min at room temperature. For the FACS analysis, the spleen cells were resuspended in Flow Cytometry Staining Buffer (eBioscience). In addition, for primary culture, the spleen cells were resuspended in RPMI 1640 (Lonza, Walkersville, MD, USA) containing 5% heat-inactivated foetal bovine serum (FBS; Gibco Laboratory, Grand Island, NY, USA), 100 U/mL penicillin, and 100 μg/mL streptomycin. Using sterile 12-well culture plates, cells were seeded at a concentration of 1 × 10^6^ in 100 μL of medium. Spleen cells were cultured for 48 h with 2.5 μg/mL Concanavalin A (Con A; Sigma-Aldrich Co.) under a humidified atmosphere of 5% CO_2_ and 95% air.

### Cytokine production of spleen cell culture supernatant

The cytokines IL-4, IL-5, IL-13, IL-17A, IL-22, and IFN-γ in the spleen cell culture supernatants were examined using the ProcartaPlex™ Immunoassay (eBioscience) according to the manufacturer’s protocol. Cytokine concentrations were measured using analyte-specific capture beads coated with target-specific analyte-specific antibodies. Following the binding of the fluorescent detection label, the reporter fluorescent signal was measured using the Luminex 200™ system and analysed using ProcartaPlex Analyst 1.0 (eBioscience). All samples and standards were measured in duplicate.

### Quantitative real time-PCR

Quantitative real time-PCR was used to evaluate cultured spleen cells. Total RNAs from cultured spleen cells were extracted using the RNeasy Mini Kit (Qiagen, Valencia, CA, USA) according to the protocol provided by the manufacturer and quantified using a NanoDrop 200 spectrophotometer (Thermo Scientific, Wilmington, DE, USA). RNA (500 ng) was reverse transcribed to obtain complementary DNA (cDNA) using the ImProm-II™ Reverse Transcription system (Promega, Madison, WI, USA) following the manufacturer’s instructions in a T-Gradient Thermoblock (Biometra, Gottingen, Germany). All TaqMan® Gene expression primers and probes for murine *IL-4, IL-5, IL-17A, IL-22, IFN-γ,* and *GAPDH* were designed by Applied Biosystems (Foster City, CA, USA) (as Inventoried Assays). The assay ID details were as follows: *IL-4* (Mm00445259_m1), *IL-5* (Mm00439646_m1), *IL-13* (Mm00434204_m1), *IL-17A* (Mm00439618_m1), *IL-22* (Mm01226722_g1), *IFN-γ* (Mm01168134_m1), and *GAPDH* (Mm99999915_g1). qRT-PCR was performed using TaqMan® Universal PCR Master mix (Applied Biosystems) and the StepOnePlus™ Real-Time PCR system (Applied Biosystems). The transcript level for each gene was normalised to that of the internal control gene *GAPDH*. Relative gene expression was acquired using the ΔΔC_t_ method, where C_t_ = threshold cycle value.

### Flow cytometric analysis

Spleen cells (1 × 10^6^ cells) isolated from mice were washed with Flow Cytometry Staining Buffer (eBioscience), stained with an FITC-conjugated anti-CD4 monoclonal antibody and APC-conjugated anti-CD25 monoclonal antibody (eBioscience), permeabilised with Fixation/Permeabilisation solution (eBioscience), and finally stained with a PE-conjugated anti-Foxp3 monoclonal antibody. After washing with Flow Cytometry Staining Buffer, the cells were analysed using a FACS Aria™ Flow Cytometer (BD Biosciences, San Jose, CA, USA). For each cell, 50,000 events were collected, and data were analysed using FACSDiva 6.1.3 (BD Biosciences).

### Western blot analysis

Spleen tissues were homogenised in RIPA buffer (Pierce Biotechnology, Rockford, IL, USA) containing protease inhibitor cocktail (Roche, Mannheim, Germany) and phosphatase inhibitor cocktail 2 and 3 (Sigma-Aldrich Co.) on ice. Then, homogenates were centrifuged at 13,400 × *g* for 20 min at 4 °C to obtain the supernatants. The protein concentrations in supernatants were measured using the Pierce BCA Protein Assay Kit (Thermo Scientific). Protein lysates (40 μg) were separated by SDS-PAGE and then transferred onto polyvinylidene difluoride (PVDF) membranes (Millipore, Billerica, MA, USA). The membranes were blocked using 5% bovine serum albumin (BSA) in Tris-buffered saline with 0.1% Tween 20 (TBS-T) for 1 h at room temperature. Primary antibodies were diluted in TSB-T containing 5% BSA and incubated overnight at 4 °C with gentle shaking. The membranes were washed three times with TBS-T and incubated at room temperature for 1 h with horseradish peroxidase-conjugated secondary antibodies in TBS-T. After three washes with TBS-T, the membranes were detected using chemiluminescent reagents (Pierce Biotechnology) according to the manufacturer’s instructions. Antibodies against calcineurin were purchased from BD Biosciences. NFAT1, 2, and secondary antibodies were purchased from ABCAM (Cambridge, UK). The anti-β-actin antibody was purchased from Santa Cruz Biotechnology (Santa Cruz, CA, USA). The intensity of bands was quantified using ImageJ, and all results were normalised to β-actin.

### Statistics

Data are expressed as means ± SD. One-sample Kolmogorov–Smirnov tests were used to evaluate the distribution characteristics of variables. Differences between groups were evaluated by one-way ANOVA and Kruskal–Wallis tests. Dunnett’s tests and Bonferroni-adjusted Mann–Whitney *U*-tests were used as post hoc tests, as appropriate. Statistical significance was accepted at *p* < 0.05. Analyses were implemented in Statistical Package for the Social Sciences (Version 23; SPSS, Chicago, IL, USA).

## Data Availability

All data generated or analysed during the current study are available from the corresponding author on reasonable request.
